# Policy optimization for high-quality development and openness of China's healthcare sector: a tripartite evolutionary game based on wholly foreign-owned hospitals, public hospitals and the government

**DOI:** 10.3389/fpubh.2025.1594684

**Published:** 2025-06-13

**Authors:** Liu Xiao, Wang Qingjin, Gao Hao, Jiang Shan

**Affiliations:** ^1^Business College, Qingdao University, Qingdao, Shandong, China; ^2^School of Foreign Languages, Qingdao University, Qingdao, Shandong, China

**Keywords:** policy optimization, China's healthcare sector, tripartite evolutionary game, wholly foreign-owned hospitals, public hospitals, government

## Abstract

**Introduction:**

With the intensification of population aging and the diversification of medical demands, China's healthcare system faces dual challenges of ensuring public welfare and improving efficiency. To enhance China's medical service capabilities, upgrade medical technologies, and expand diversified service supply patterns, the Chinese government has permitted the establishment of wholly foreign-owned hospitals.

**Methods:**

This study constructs a tripartite evolutionary game model involving wholly foreign-owned hospitals, public hospitals, and the government, combined with system dynamics simulation, to explore policy optimization pathways for high-quality development and sustained opening-up in China's healthcare industry.

**Results and discussion:**

The entry decisions of wholly foreign-owned hospitals are driven by economic returns and policy support, where government subsidies can accelerate their entry into the Chinese market but exhibit diminishing marginal utility. The transformation of public hospitals is primarily influenced by government financial subsidies, with subsidy intensity exhibiting a positive correlation with the degree of transformation; however, high subsidies are unsustainable. Government regulatory strategies are constrained by both social losses and regulatory costs, which require balancing short-term interventions with long-term sustainability. Based on these findings, this study argues that China's healthcare industry should continuously improve the regulations on foreign investment access, allow social funds to enter the healthcare industry, establish comprehensive urban medical alliances, and refine the regulatory measures, among others.

## 1 Introduction

Since the founding of the People's Republic of China, the country's healthcare sector has achieved remarkable progress (1). Since the launch of reform and opening-up, the Chinese government has established a nearly universal healthcare insurance system, significantly enhancing the efficiency of medical services (2). However, with the deepening of China's population aging, public demands for hospital services are growing in both scale and quality. But, China's public hospitals, having long shouldered basic medical needs (3), face challenges such as insufficient supply of high-end medical services and inadequate momentum for transformation. Therefore, to achieve high-quality development in the healthcare sector and accelerate the establishment of a modern hospital management system, ensuring access to diverse healthcare services for both China's residents and foreign nationals in China, the Chinese government has decided to fully lift foreign investment access restrictions in the healthcare field (4), permitting the establishment of wholly foreign-owned hospitals in nine regions, including Beijing, Tianjin, and Shanghai (5).

The entry of wholly foreign-owned hospitals will effectively drive the China's healthcare system toward diversified and personalized development, meeting multi-tiered demands and addressing gaps in high-end medical services (6). These hospitals exhibit greater flexibility in drug access, enabling them to provide patients with imported or originator drugs not yet available in China (7). Moreover, wholly foreign-owned hospitals can introduce mature modern hospital management philosophies, efficient governance models, and refined service practices, thereby promoting the optimization of service processes in China's medical institutions. They will also stimulate the development of upstream and downstream industries, fostering the formation of regional healthcare industry clusters (8). This will further elevate China's status and influence in the global healthcare sector, enhance international collaboration and exchanges in the healthcare field, and advance the growth of China's medical service trade.

As China's healthcare system has always adhered to the public welfare principle, public hospitals are required to consistently provide equitable and accessible medical services that benefit the public interest, rather than pursuing self-serving goals or prioritizing institutional or individual gains (9). However, since the launch of the new healthcare reform, the rapid expansion of public hospitals has led to escalating financial burdens, excessive growth in healthcare costs, and severe imbalances in the distribution of basic social medical insurance funds (10). Consequently, it is imperative to deepen the transformation of public hospitals, build high-caliber public healthcare institutions, and promote their high-quality development (11).

The Chinese government's decision to permit wholly foreign-owned hospitals to enter the China's healthcare sector fundamentally aims to establish a complementary structure alongside public hospitals, while leveraging competitive pressures to compel proactive transformation of public hospitals and stimulate industry competition (12). Since the new healthcare reform, China has made progress in expanding insurance coverage and introducing social capital into healthcare. However, as the main body of medical services, public hospitals continue to grapple with inefficiencies and resource misallocation (13). Therefore, by introducing the “catfish effect” through wholly foreign-funded hospitals, China seeks to establish market competition mechanisms that enhance healthcare system efficiency, thereby driving public hospitals to further optimize service quality and operational effectiveness.

However, divergent objectives among wholly foreign-owned hospitals, public hospitals, and the government inevitably generate multi-stakeholder conflicts of interest during China's pursuit of high-quality development and openness in healthcare. Moreover, the decision-making behaviors of these stakeholders are demonstrably influenced by subjective cognitive biases and conflicting values. Specifically, what factors shape each stakeholder's strategic choices? What evolutionary trends emerge from these factors? How can optimal evolutionary pathways be identified? How can stakeholders be guided toward an optimal equilibrium state? The key to solving these problems lies in clarifying the conflicts of interest among stakeholders and revealing the evolutionary laws of the dynamic evolutionary game paths of stakeholders.

The decision-making processes of wholly foreign-owned hospitals, public hospitals, and the government are shaped by costs, benefits, losses, government subsidies, and the decisions of other stakeholders. Therefore, it is critical to investigate how these stakeholders are influenced by these variables. An evolutionary game analysis of China's high-quality healthcare development comprehensively incorporates the strategic evolutionary processes of all stakeholders, who act rationally to minimize costs and maximize their benefits (14). To better align with real-world dynamics, this study constructs a tripartite evolutionary game model that integrates the major stakeholders in China's healthcare sector—wholly foreign-owned hospitals, public hospitals, and the government—into a unified analytical framework, reconciling their divergent interests. Furthermore, adopting evolutionary game theory and simulation analysis as novel approaches, the research examines the evolutionary trajectories of stakeholder strategies and shifts in payoffs under varying conditions. This methodology precisely delineates the roles of foreign-funded hospitals, public hospitals, and the government in advancing high-quality development within China's healthcare industry. These findings enhance the rigor of the analysis, determine the optimal strategic equilibrium for all stakeholders, and provide practical guidance for policy implementation.

## 2 Literature review

### 2.1 Research on wholly foreign-owned hospitals

Most scholars argue that wholly foreign-owned hospitals and public hospitals, given their distinct roles within the social healthcare system, exhibit both competitive and complementary relationships, constituting a vital component of the medical treatment combination (15). Moreover, the entry of wholly foreign-owned hospitals can drive reforms in China's healthcare sector enhance the quality of medical services, and improve physicians' income structures. Hence, extensive research has been conducted by scholars to evaluate the advantages and disadvantages of wholly foreign-owned hospitals entering China.

In terms of functionality, scholars have integrated that wholly foreign-owned hospitals stimulate market competition and innovation. Wholly foreign-owned hospitals equipped with advanced international medical technologies and overseas professional teams, address China's technological gaps and compelling China's healthcare institutions to align with global standards (16). In terms of management, wholly foreign-owned hospitals entry compels public hospitals to streamline service processes and enhance efficiency (17), while driving innovation in hospital management philosophies and research collaboration. This objectively elevates the service quality and operational efficiency of public hospitals, serving as a critical measure to rationalize the allocation of China's healthcare resources (18). Additionally, the growth of wholly foreign-owned hospitals contributes to weakening the traditional administrative hierarchy between the government and hospitals, prompting stricter regulatory oversight over medical institutions (19). In terms of social benefits, wholly foreign-owned hospitals also accelerate the development of China's modern healthcare insurance sector, fostering a commercial health insurance market that prioritizes personalization and segmentation, thereby solidifying commercial insurance as a pillar of the social security system (20). Although wholly foreign-owned hospitals may lure core medical talents from public hospitals through competitive salaries and career advancement opportunities, in the long run, such mobility could incentivize public hospitals to optimize compensation systems and improve professional environments, ultimately enhancing the healthcare sector's overall appeal (21).

However, the entry of wholly foreign-owned hospitals also poses challenges for China's public hospitals. The entry of wholly foreign-owned hospitals may intensify challenges related to healthcare resource allocation equity, particularly in balancing accessibility between premium services for high-income groups and universal basic healthcare guarantees (22). The uneven distribution of high-end medical resources forces patients to seek care across regions to access higher-quality medical services (23). Moreover, premium medical services (such as imported medical devices and personalized care) remain predominantly financed through self-payment and commercial insurance coverage, potentially exacerbating socioeconomic stratification in healthcare access (24). Public hospitals, operating on razor-thin profit margins, face mounting financial pressures from multiple fronts: uncompensated care for uninsured patients, rising costs of supplies and technologies, and escalating labor expenses (25). Concurrently, public hospitals in remote rural areas often struggle with low patient volumes, making it difficult to attract and retain medical staff, which has led to closures of rural facilities (26). This vicious cycle further entrenches geographic and socioeconomic disparities in healthcare resource allocation.

Researchers have conducted extensive studies on the entry of wholly foreign-owned hospitals into China. Most of this research highlights the positive impacts, such as promoting technological advancements in China's healthcare, optimizing the medical industry, and improving the management capabilities of public hospitals through increased competition. However, it has also been noted that wholly foreign-owned hospitals can lead to issues like an excessive concentration of medical resources and rising healthcare costs for residents. Despite these concerns, scholars generally support the introduction of wholly foreign-funded hospitals. These research findings indicate that wholly foreign-owned hospitals can serve as a beneficial driving force for the reform and development of China's healthcare sector. Nonetheless, practical implementation faces challenges. To ensure a smooth entry and effective operation of these hospitals while minimizing negative impacts, it is essential to develop sound policies and management measures.

### 2.2 Research on the transformation of public hospitals

As the backbone of China's healthcare system, public hospitals have long shouldered the public welfare mandate of providing essential medical services (9). However, with intensifying population aging, diversifying healthcare demands, and evolving policy landscapes, they now face multifaceted challenges including efficiency bottlenecks, resource misallocation, and operational strain (27). In recent years, amid the deepening healthcare reform and the entry of wholly foreign-owned hospitals, the transformation of public hospitals has emerged as a focal point in both academic and policy circles (28).

Some scholars argue that the core of the transformation of public hospitals lies in optimizing internal operational mechanisms and service delivery models to enhance healthcare capabilities (29). Firstly, most researchers emphasize the need to systematically reform governance structures by centralizing the authority for healthcare policy formulation and public hospital supervision under a unified committee, enabling comprehensive management of all public hospitals (30). Secondly, through national policies such as the “zero markup drug policy” and centralized drug procurement, public hospitals should eliminate financial reliance on pharmaceutical sales (31). Thirdly, public hospitals must fully integrate market competition mechanisms into physician compensation structures, fundamentally reforming salary systems to align incentives with service quality and efficiency (32).

To address the challenges of wholly foreign-owned hospitals, transforming public hospitals requires integrating external governance policies with internal management measures, shifting from rapid scale expansion to high-quality development of service capabilities (33). Public hospitals should abandon traditional independent expansion practices and establish interdisciplinary entities (e.g., collaborations in medical engineering, medical science, and medical humanities). They should adopt strategies such as multi-campus hospital models, professional alliances (34), and medical consortia to integrate resources both within individual hospitals and across multiple campuses. This facilitates the formation of regional or cross-regional healthcare networks, advancing the specialization of public hospitals and the centralization of specialized services (35). Simultaneously, the transformation of public hospitals also lies in the extensive application of technologies such as digitalization and artificial intelligence. By leveraging big data and patient data to conduct personalized assessments of patients' symptoms, public hospitals can enhance their capabilities in health management, diagnosis, and treatment (36).

However, scholars note that while the transformation of public hospitals has reshaped their revenue structure, reducing reliance on pharmaceutical income and increasing service-based revenue (37), it has also partially increased government healthcare expenditures due to the unique institutional nature of public hospitals (38). Moreover, excessive and disorderly competition may squeeze fiscal expenditures on technology adoption and medical services, ultimately hindering the improvement of medical service quality in public hospitals.

In the research on the transformation of public hospitals, most of the existing studies explore the necessity and advantages of the transformation of public hospitals from a theoretical perspective. Moreover, many scholars tacitly assume that public hospitals will inevitably choose to transform when faced with wholly foreign-owned hospitals. However, these studies rarely include an analysis based on the actual revenue situation of public hospitals. Therefore, it is necessary to combine theory with practice, explore the factors and paths that affect the transformation of public hospitals, and enhance the motivation for the transformation of public hospitals.

### 2.3 Research on the government regulation

Due to the healthcare sector exhibits information asymmetry, externalities, and public good attributes, making exclusive reliance on market mechanisms prone to market failures such as induced demand, over-treatment, and uneven resource distribution (39). Wholly foreign-owned hospitals and public hospitals, as two core pillars of China's public healthcare service system, require a robust regulatory framework to ensure efficient resource allocation, service quality, and social equity (25). Studies widely assert that governments must balance efficiency with equity through multi-dimensional interventions, including access regulation, quality oversight, price controls, and public subsidy governance (40).

The primary objective of government regulation is to address healthcare sector deficiencies and safeguard the equity and accessibility of healthcare services (19). Therefore, in the process of deepening the reform of the medical system, China's government regulatory model is transitioning from traditional administrative mandates toward diversified and refined approaches, characterized by shifts from fragmented to systematic governance and from administrative intervention to rule-of-law-based oversight (29). It mainly includes the government's formulation of development plans and relevant policies for the medical and health undertakings to guide hospitals to strengthen primary medical services (41). This prevents excessive marketization or commercialization, ensuring alignment with the overarching strategy of healthcare system development and safeguarding the equity and accessibility of medical services (42). Secondly, government oversight guarantees that institutional reforms, such as adjustments to property rights structures, innovations in management systems, and shifts in service delivery models, are implemented by laws and regulations. This ensures fairness and transparency throughout the transformation of public hospitals, as well as the compliance of wholly foreign-owned hospitals with relevant regulations in China (43). Third, government regulation facilitates the optimized integration of medical resources between wholly foreign-owned hospitals and public hospitals. By fostering complementary strengths between these institutions, advancing the development of medical consortia, strengthening collaboration between foreign-funded hospitals and local healthcare providers, and encouraging social capital participation in public hospital transformation, thereby improving resource efficiency and reducing waste (44).

### 2.4 Research on the evolutionary game theory

The focus of the evolutionary game theory was on the dynamics of strategy change as influenced by the various competing systems in different situations of game (45). Its theory originates from the long-term interaction of competitive behaviors in the process of biological evolution (46). Therefore, the evolution process of the stakeholder behavior can be comprehensively and accurately analyzed when the influencing factors change. The biggest feature of the evolutionary game is that the stakeholders can select optimal strategies to maximize their own payoffs based on the outcomes of various strategic choices. The foundational premise of game theory incentivizes stakeholders to prioritize self-interest maximization without considering others' payoffs, leading to the emergence of a Nash equilibrium (47). However, in reality, stakeholders may adapt their decisions based on perceived changes in costs, risks, and contextual dynamics. Evolutionary game theory not only reveals behavioral patterns and strategic selection mechanisms of stakeholders but also predicts the interactive effects of competing strategies, offering insights into systemic outcomes (48).

Against the backdrop of the bounded rationality of participants in the healthcare industry and information asymmetry, evolutionary game theory has also been widely applied in medical decision-making in recent years. Based on the complexity of the real healthcare industry environment, many studies tend to use a variety of game models to simulate the behaviors of participants in the healthcare industry. Some scholars have analyzed the behaviors of individuals in the interactive model of disease vaccination based on the evolutionary game approach. This approach captures the impact of vaccination decisions on the prevalence of diseases, including social learning (49). To optimize hospital medical supply chain management, researchers have constructed tripartite evolutionary game models involving hospitals, service providers, and suppliers. These models are further integrated with system dynamics simulations to inform decision-making under the Supply-Processing-Distribution (SPD) model (50). Other scholars developed a tripartite evolutionary game model involving China's pharmaceutical companies, foreign pharmaceutical firms, and governments to analyze strategic choices under China's centralized volume-based procurement system (51). Other scholars built a “government-hospital” evolutionary game model to study the evolution of medical information-sharing behaviors among hospitals under regulatory oversight (52). Some scholars incorporated governments, hospitals, and patients into a unified framework to investigate how regulatory interventions shape strategy evolution in doctor-patient disputes (53). There are also scholars who explored rent-seeking behaviors between pharmaceutical manufacturers and third-party drug testing agencies through a tripartite evolutionary game model involving enterprises, regulators, and governments (54). These studies demonstrate how evolutionary game theory provides a robust analytical lens for decoding strategic interactions, policy impacts, and systemic equilibria in complex healthcare systems.

In summary, there is a broad scholarly consensus that the entry of wholly foreign-owned hospitals and the transformation of public hospitals have together driven the opening up and reform of China's healthcare system. Wholly foreign-owned hospitals have a significant impact through technology spillovers and service upgrades; however, caution is necessary to avoid imbalances in resource allocation and ethical risks. Reforms in public hospitals need to balance their mandate for public welfare with the necessity for operational efficiency, achieving high-quality development through institutional innovations, and technological empowerment. Meanwhile, the government must employ policy tools to address systemic gaps while ensuring that the healthcare system remains equitable and prioritizes public welfare.

Nonetheless, existing research predominantly remains theoretical, with limited exploration of the dynamic decision-making logics among wholly foreign-owned hospitals, public hospitals, and the government, the interplay of influencing factors, and mechanisms to achieve optimal strategic equilibria while ensuring long-term stability. To address these gaps, this study introduces a tripartite evolutionary game framework with the following objectives: assessing strategic stability among stakeholders by constructing replicator dynamic equations; conducting stability analysis on equilibrium points; and identifying evolutionarily stable strategy combinations under varying conditions. The findings will be validated through simulation-based scenarios, quantifying the effects of key parameters. The results will offer actionable policy recommendations to optimize the mechanisms for foreign hospital entry, accelerate the transformation of public hospitals, and refine multi-tiered regulatory systems, ultimately advancing China's healthcare sector toward equitable, efficient, and sustainable development.

## 3 Model hypothesis and construction

Hypothesis 1: wholly Foreign-owned hospitals, public hospitals, and the government are bounded rational participants that prioritize maximizing their own interests. Their strategic choices are influenced by factors such as access to information, decision-making preferences, and contextual constraints, all participants in the game can gradually stabilize the strategy choices to the optimal strategy through continuous trial and error and learning (55).
Hypothesis 2: based on their analysis of the current development status and future trends in China's healthcare sector, wholly foreign-owned hospitals can choose to enter the China's healthcare sector or not. Their strategic choices are {Enter, Not Enter}, with a probability of *x* for choosing to enter. In response to patient demand for high-quality healthcare services, public hospitals can choose to proactively transform to meet market needs or maintain the status quo. Their strategic choices are {Transform, Not Transform}, with a probability of *y* for choosing transformation. The government can adopt policy regulation, utilizing measures such as legislation and financial subsidies to support wholly foreign-owned hospitals' entry into China and encourage public hospitals to proactively transform. Alternatively, it may refrain from regulation and not intervene with in the strategic choices of wholly foreign-owned and public hospitals. The government's strategic choices are {Regulate, Not Regulate}, with a probability of *z* for choosing regulation, 0 ≤ *x, y, z* ≤ 1.
Hypothesis 3: when wholly foreign-owned hospitals choose to enter the China's healthcare sector, they can obtain additional operational revenue *R*_1_ due to the substantial demand for high-end foreign medical services in China (56). Furthermore, by introducing cutting-edge pharmaceuticals, technologies, and equipment to deliver higher-quality medical services, along with hospital environments that prioritize patient comfort and experience, they gain favorable reputation benefits *S*_1_ (18). This enhanced reputation helps attract more patients. However, hospital development typically involves substantial capital investment, extended payback periods, and significant investment risks. Wholly foreign-owned hospitals must bear upfront costs *C*_1_ for infrastructure construction, staff recruitment, and training, among other initial expenses, which can be reduced with government support (16). If wholly foreign-owned hospitals choose not to enter the China's healthcare sector, they will neither incur additional costs nor obtain related benefits.
Hypothesis 4: when public hospitals choose to transform, they must optimize institutional management and improve service quality, thereby incurring transform costs *C*_2_ for hardware upgrades, staff training, and other related expenses. However, this transformation enables them to attract more patients, generating additional operational benefits *R*_2_ (50). Conversely, if public hospitals opt not to transform, they will face reputational loss *S*_2_ due to outdated equipment, poor patient experience, and similar factors (43). Additionally, if wholly foreign-funded hospitals enter China's healthcare sector under such circumstances, public hospitals burdened by their poor reputation, must contend with competitive losses *C*_3_ arising from heightened competition, staff attrition, and patient outflow (57).
Hypothesis 5: when the government chooses to regulate, it incurs a fixed regulatory cost *C*_4_ to oversee the quality, safety, operational compliance, and technology access of wholly foreign-owned hospitals (58). Additionally, the government bears a variable cost of *R*_3_ to provide financial subsidies to public hospitals actively pursuing transformation, thereby incentivizing their transformation efforts. Simultaneously, the government offers policy support to qualified foreign-owned hospitals that meet entry requirements, such as streamlining approval procedures and recruitment processes, to reduce their entry costs *C*_5_ (37). When foreign-funded hospitals enter China's healthcare industry or public hospitals choose to carry out reforms, the government will obtain additional social benefits *R*_4_, such as more tax revenues and greater social credibility because patients will have more options for seeking medical treatment and a better medical environment (59). If the government chooses not to regulate, it can avoid direct cost expenditures but may suffer societal losses *S*_3_ due to potential social risks, such as excessive pricing or unethical billing practices by foreign-owned hospitals (22), as well as systemic inefficiencies caused by stagnation in public hospitals (26). (Table 1)

**Table 1:** The basic variables in the game model.

**Participant**	**Parametric**	**Meaning of parameters**
Wholly foreign-owned hospitals	*R*_1_	Additional operational benefits when choosing to enter China
	*C*_1_	Construction costs when choosing to enter China
	*S*_1_	Reputational benefits to be gained when choosing to enter China
Public hospitals	*R*_2_	Additional benefits of choosing to transform
	*C*_2_	Additional cost of choosing to transform
	*S*_2_	Reputational losses from choosing not to transform
	*C*_3_	Competitive losses of public hospitals that chose not to transform when foreign hospitals entered China
Government	*C*_4_	Costs of choosing regulation
	*C*_5_	Entry costs of wholly foreign-owned hospitals reduced by policy support when government chooses to regulate
	*R*_3_	Financial subsidies for the transformation of public hospitals when the Government chooses to regulate
	*R*_4_	Additional social benefits to the Government when Wholly Foreign-owned hospitals choose to enter or public hospitals choose to transform
	*S*_3_	Social losses when governments choose not to regulate

Based on the above assumptions, a tripartite game payoffs matrix is constructed, as shown in Table 2.

**Table 2:** Tripartite game payoffs matrix.

**Combination of strategies**			**Wholly foreign-owned hospitals**	**Public hospitals**	**Government**
Enter	Transform	Regulate	*R*_1_−*C*_1_+*S*_1_+*C*_5_	*R*_2_−*C*_2_+*R*_3_	*R*_4_−*C*_4_−*R*_3_
Enter	Transform	Not regulate	*R*_1_−*C*_1_+*S*_1_	*R*_2_−*C*_2_	−*S*_3_
Enter	Not transform	Regulate	*R*_1_−*C*_1_+*S*_1_+*C*_5_	−*C*_3_−*S*_2_	*R*_4_−*C*_4_
Enter	Not transform	Not regulate	*R*_1_−*C*_1_+*S*_1_	−*C*_3_−*S*_2_	−*S*_3_
Not enter	Transform	Regulate	0	*R*_2_−*C*_2_+*R*_3_	*R*_4_−*C*_4_−*R*_3_
Not enter	Transform	Not regulate	0	*R*_2_−*C*_2_	−*S*_3_
Not enter	Not transform	Regulate	0	−*S*_2_	−*C*_4_
Not enter	Not transform	Not regulate	0	−*S*_2_	−*S*_3_

## 4 Evolutionary game and its strategy stability analysis

The replication dynamic equations for each participant's strategy choice can be obtained based on the benefit matrix of the tripartite game between wholly foreign-owned hospitals, public hospitals, and the government.

### 4.1 Strategy stability analysis of wholly foreign-owned hospitals

The expected payoffs of wholly foreign-owned hospitals choosing to enter or not enter China are denoted as $E_{1}^{\left(1\right)}$ and $E_{1}^{\left(2\right)}$, respectively, and the average prospect value is represented by Ē_1_:


(1) $$\begin{array}{l} E_{1}^{\left(1\right)}=z[y(R_{1}-C_{1}+S_{1}+C_{5})+\left(1-y\right)(R_{1}-C_{1}+S_{1}+C_{5})]\;\\ \;+\;\left(1-z\right)\left[y\left(R_{1}-C_{1}+S_{1}\right)+\left(1-y\right)\left(R_{1}-C_{1}+S_{1}\right)\right] \end{array}$$

(2) $$\begin{array}{lll} E_{1}^{\left(2\right)} & = & 0 \end{array}$$

(3) $${\bar{E}}_{1}=xE_{1}^{\left(1\right)}+(1-x)E_{1}^{\left(2\right)}$$

The replicated dynamic equation can be calculated as follows:


(4) $$\begin{array}{l} \;F\left(x\right)=\frac{dx}{dt=}x\left(E_{1}^{\left(1\right)}-{\bar{E}}_{1}\right)=x\left(1-x\right)\\ \left(E_{1}^{\left(1\right)}-E_{1}^{\left(2\right)}\right)=x(1-x)(R_{1}+S_{1}-C_{1}+zC_{5}) \end{array}$$

Find the first derivative of Equation 4:


$$\begin{array}{l} \frac{dF\left(x\right)}{dx}=\left(1-2x\right)\left(R_{1}+S_{1}-C_{1}+zC_{5}\right) \end{array}$$

Set *G*(z^*^) to:


$$G(z^{*})=R_{1}+S_{1}-C_{1}+zC_{5}$$

According to the stability theorem of differential equations, the following condition must be satisfied for wholly foreign-owned hospitals to reach a steady state in their choosing to enter the China: *F*(*x*) = 0 and $\frac{dF\left(x\right)}{dx}<0$. Let *G*(z^*^) = 0, then:


$$z=z^{*}=\frac{C_{1}-R_{1}-S_{1}}{C_{5}}$$

When *z*>z^*^, *G*(*z*)>0, *F*(1) = 0, and ${\frac{dF\left(x\right)}{dx}}_{x=1}<0$. In this case, *x* = 1 is an evolutionarily stable strategy for wholly foreign-owned hospitals that choose to enter China. When *z* = z^*^, *G*(*z*) = 0, *F*(*x*) = 0, and $\frac{dF\left(x\right)}{dx}\equiv 0$, all values of *x* are in equilibrium, i.e., the strategy adopted by wholly foreign-owned hospitals does not effect the evolutionary stability regardless of whether they choose to enter or not enter China. When *z*<z^*^, *G*(*z*) < 0, *F*(0) = 0, and ${\frac{dF\left(x\right)}{dx}}_{x=0}<0$, *x* = 0 is an evolutionarily stable strategy for wholly foreign-owned hospitals indicating a strategy of not entering China. In summary, the probability of wholly foreign-owned hospitals choosing to enter China increases from *x* = 0 to *x* = 1 as *z* gradually increases.

### 4.2 Strategy stability analysis of public hospitals

The expected payoffs of public hospitals choosing to transform or not transform are denoted as $E_{2}^{\left(1\right)}$ and $E_{2}^{\left(2\right)}$, respectively, and the average prospect value is represented by Ē_2_:


(5) $$\begin{array}{l} E_{2}^{\left(1\right)}=z\left[x(R_{2}-C_{2}+R_{3})+\left(1-x\right)(R_{2}-C_{2}+R_{3})\right]+\;(1-z)\\ \;[x(R_{2}-C_{2})+(1-x)(R_{2}-C_{2})] \end{array}$$

(6) $$\begin{array}{l} E_{2}^{\left(2\right)}=z[x\left(-C_{3}-S_{2}\right)-(1-x)S_{2}]+\left(1-z\right)[x\left(-C_{3}-S_{2}\right)\\ \;-(1-x)S_{2}] \end{array}$$

(7) $${\bar{E}}_{2}=yE_{2}^{\left(1\right)}+(1-y)E_{2}^{\left(2\right)}$$

The replicated dynamic equation can be calculated as follows:


(8) $$\begin{array}{l} F\left(y\right)=\frac{dy}{dt=}y\left(E_{2}^{\left(1\right)}-{\bar{E}}_{2}\right)=y\left(1-y\right)\left(E_{2}^{\left(1\right)}-E_{2}^{\left(2\right)}\right)\\ \;=y\left(1-y\right)(R_{2}-C_{2}+S_{2}+xC_{3}-zR_{3}) \end{array}$$

Find the first derivative of Equation 8:


$$\begin{array}{l} \frac{dF\left(y\right)}{dy}=\left(1-2y\right)\left(R_{2}-C_{2}+S_{2}+xC_{3}-zR_{3}\right) \end{array}$$

Set *M*(x^*^) to:


$$\begin{array}{l} M\left(x^{*}\right)=R_{2}-C_{2}+S_{2}+xC_{3}-zR_{3} \end{array}$$

Similarly, let *M*(x^*^) = 0, then:


$$\begin{array}{l} x=x^{*}=\frac{zR_{3}-S_{2}-R_{2}+C_{2}}{C_{3}} \end{array}$$

When *x*>x^*^, *M*(*x*)>0, *F*(1) = 0, and ${\frac{dF\left(y\right)}{dy}}_{y=1}<0.$ In this case, *y* = 1 is an evolutionarily stable strategy for public hospitals that choose to transform. When *x* = x^*^, *M*(*x*) = 0, *F*(*y*) = 0, and $\frac{dF\left(y\right)}{dy}\equiv 0$, all values of *y* are in equilibrium; i.e., the strategy adopted by public hospitals does not effect evolutionary stability regardless of whether they transform or not. When *x*<x^*^, *M*(*x*) < 0, *F*(0) = 0, and ${\frac{dF\left(y\right)}{dy}}_{y=0}<0$, *y* = 0 is an evolutionarily stable strategy for public hospitals, indicating a choice not to transform. In summary, as *x* increases, the probability of the public hospitals choosing to transform strategy increases from *y* = 0 to *y* = 1.

### 4.3 Strategy stability analysis of government

The expected payoffs of the government choosing to regulate or not to regulate are denoted as $E_{3}^{\left(1\right)}$ and $E_{3}^{\left(2\right)}$, respectively, and the average prospect value is represented by Ē_3_:


(9) $$\begin{array}{l} E_{3}^{\left(1\right)}=x\left[y\left(R_{4}-C_{4}-R_{3}\right)+\left(1-y\right)\left(R_{4}-C_{4}\right)\right]+\left(1-x\right)\\ \;[y\left(R_{4}-C_{4}-R_{3}\right)-\left(1-y\right)C_{4}] \end{array}$$

(10) $$\begin{array}{l} E_{3}^{\left(2\right)}=x\left[y(-S_{3})+\left(1-y\right)(-S_{3})\right]+\left(1-x\right)[y\left(-S_{3}\right)\\ \;-\left(1-y\right)S_{3}] \end{array}$$

(11) $${\bar{E}}_{3}=zE_{3}^{\left(1\right)}+(1-z)E_{3}^{\left(2\right)}$$

The replicated dynamic equation can be calculated as follows:


(12) $$\begin{array}{l} F\left(z\right)=\frac{dz}{dt=}z\left(E_{3}^{\left(1\right)}-{\bar{E}}_{3}\right)=z\left(1-z\right)\left(E_{3}^{\left(1\right)}-E_{3}^{\left(2\right)}\right)=z\left(1-z\right)\\ \;(S_{3}-C_{4}+xR_{4}-yR_{3}) \end{array}$$

Find the first derivative of Equation 12:


$$\begin{array}{l} \frac{dF\left(z\right)}{dz}=\left(1-2z\right)\left(S_{3}-C_{4}+xR_{4}-yR_{3}\right) \end{array}$$

Set *J*(y^*^) to:


$$\begin{array}{l} J\left(y^{*}\right)=S_{3}-C_{4}+xR_{4}-yR_{3} \end{array}$$

Similarly, let *J*(y^*^) = 0, then:


$$\begin{array}{l} y=y^{*}=\frac{S_{3}-C_{4}+xR_{4}}{R_{3}} \end{array}$$

When *y*>y^*^, *J*(*y*)>0, *F*(1) = 0, and ${\frac{dF\left(z\right)}{dz}}_{z=1}<0$. In this case, *z* = 1 is an evolutionarily stable strategy for the government, indicating a choice to regulate. When *y* = y^*^, *M*(*y*) = 0, *F*(*z*) = 0, and $\frac{dF\left(z\right)}{dz}\equiv 0$, all values of *z* are in equilibrium, i.e., the strategy adopted by the government does not effect evolutionary stability regardless of whether it regulates or not. When *y*<y^*^, *J*(*y*) < 0, *F*(0) = 0, and ${\frac{dF\left(z\right)}{dz}}_{z=0}<0$, *z* = 0 is an evolutionarily stable strategy for the government, indicating a choice not to regulate. In summary, the probability of the government choosing to regulate increases from *z* = 0 to *z* = 1 as *y* gradually increases.

### 4.4 Equilibrium point and stability analysis of an evolutionary game system

In order to provide a more comprehensive analysis of the equilibrium strategies of the tripartite evolutionary game system, a system of replicated dynamic Equation 13 has been developed based on Equations 4, 8, 12.


(13) $$\left\{ \begin{array}{l} \;F\left(x\right)=x\left(1-x\right)\left(R_{1}+S_{1}-C_{1}+zC_{5}\right)\\ F\left(y\right)=y\left(1-y\right)(R_{2}-C_{2}+S_{2}+xC_{3}-zR_{3})\\ \;F\left(z\right)=z\left(1-z\right)(S_{3}-C_{4}+xR_{4}-yR_{3}) \end{array}\right.$$

Let *F*(*x*) = 0, *F*(*y*) = 0, *F*(*z*) = 0, then *S*_1_(0, 0, 0), *S*_2_(0, 1, 0), *S*_3_(1, 0, 0), *S*_4_(0, 0, 1), *S*_5_(1, 1, 0), *S*_6_(1, 0, 1), *S*_7_(0, 1, 1), and *S*_8_(1, 1, 1), are strategic partial equilibrium points for tripartite evolutionary game system.

Friedman (60) argued that the evolutionary stability strategy could be obtained by stability analysis of the system's Jacobian matrix. According to Equation 13, the Jacobian matrix can be expressed as follows:


$$\begin{array}{l} J=\left[\begin{array}{c} \frac{dx/dt}{dx}\;\frac{dx/dt}{dy}\;\frac{dx/dt}{dz}\\ \frac{dy/dt}{dx}\;\frac{dy/dt}{dy}\;\frac{dy/dt}{dz}\\ \frac{dz/dt}{dx}\;\frac{dz/dt}{dy}\;\frac{dz/dt}{dz} \end{array}\right] \end{array}$$

According to the first theorem of Lyapunov, it is known that this equilibrium point is evolutionarily stable if all three eigenvalues are negative (61). The eight equilibrium points of the system are brought into the Jacobi matrix to calculate the Eigenvalues of each equilibrium point and its evolutionary stability conditions, as shown in Table 3.

**Table 3:** The stability analysis of the equilibrium point.

**Equilibrium point**	**λ_1_**	**λ_2_**	**λ_3_**
*E*_1_(0, 0, 0)	*R*_1_−*C*_1_+*S*_1_	*R*_2_−*C*_2_+*S*_2_	*S*_3_−*C*_4_
*E*_2_(0, 1, 0)	*R*_1_−*C*_1_+*S*_1_	*C*_2_−*R*_2_−*S*_2_	*S*_3_−*C*_4_−*R*_3_
*E*_3_(1, 0, 0)	*C*_1_−*R*_1_−*S*_1_	*C*_3_−*C*_2_+*R*_2_+*S*_2_	*R*_4_−*C*_4_+*S*_3_
*E*_4_(0, 0, 1)	*R*_1_−*C*_1_+*S*_1_+*C*_5_	*C*_3_−*C*_2_+*R*_2_+*S*_2_	*C*_4_−*S*_3_
*E*_5_(1, 1, 0)	*C*_1_−*R*_1_−*S*_1_	_*C*_2_−*C*3_−*R*_2_−*S*_2_	*R*_4_−*C*_4_−*R*_3_+*S*_3_
*E*_6_(1, 0, 1)	*C*_1_−*R*_1_−*S*_1_−*C*_5_	*C*_3_−*C*_2_+*R*_2_+*S*_2_+*R*_3_	*C*_4_−*R*_4_−*S*_3_
*E*_7_(0, 1, 1)	*R*_1_−*C*_1_+*S*_1_+*C*_5_	_*C*_2_−*C*3_−*R*_2_−*S*_2_	*C*_4_+*R*_3_−*S*_3_
*E*_8_(1, 1, 1)	*C*_1_−*R*_1_−*S*_1_−*C*_5_	*C*_2_−*C*_3_−*R*_2_−*S*_2_−*R*_3_	*C*_4_+*R*_3_−*R*_4_−*S*_3_

The current Chinese government strategy choice aims to incentivize the entry of wholly foreign-owned hospitals into the China's healthcare sector, leveraging their presence to create a “catfish effect” that stimulates systemic reforms in the healthcare sector, elevates the quality of healthcare services, and optimizes the income structure for medical professionals. Therefore, this study mainly investigates the policy intervention of the government in the entry of wholly foreign-owned hospitals enter to China and the transformation of public hospitals. Four equilibrium points, *E*_4_(0, 0, 1), *E*_6_(1, 0, 1), *E*_7_(0, 1, 1), and *E*_8_(1, 1, 1), are mainly selected for discussion.

Scenario 1: when *R*_1_+*S*_1_<*C*_1_−*C*_5_, *S*_2_+*C*_3_<*C*_2_−*R*_2_, and *C*_4_<*S*_3_ hold, i.e., the overall benefits of wholly foreign-owned hospitals entering the China's healthcare sector are less than their costs, the costs of public hospital transformation exceed the losses from not transform, and the social loss of government not regulation exceeds regulatory costs. The corresponding stable equilibrium point is *S*_7_(0, 0, 1), indicating the evolutionarily stable strategy combination as (not enter, not transform, and regulation). In this scenario, since the social loss caused by not regulation surpasses regulation costs, the government will choose regulation. However, given that wholly foreign-owned hospitals' net benefits from market entry remain insufficient and public hospitals face relatively minor losses without transformation, even under government regulation, wholly foreign-owned hospitals ultimately opt against entering the China's healthcare sector. In contrast, public hospitals do not transform.

Scenario 2: when *C*_1_−*C*_5_<*R*_1_+*S*_1_, *C*_3_+*S*_2_<*C*_2_−*R*_2_−*R*_3_, and *C*_4_−*R*_4_<*S*_3_ hold, i.e., the overall benefits of wholly foreign-owned hospitals entering the China's healthcare sector are exceeding than their costs, the costs of public hospital transformation exceed the losses from not transform, and the social loss of government not regulation exceeds regulatory costs. The corresponding stable equilibrium point is *E*_6_(1, 0, 1), indicating the evolutionarily stable strategy combination as (enter, not transform, and regulation). In this scenario, since the social loss from not regulation exceeds the comprehensive costs and benefits of regulation, the government will adopt regulation. Meanwhile, wholly foreign-owned hospitals' entry costs under government support become lower than their overall benefits, yet public hospitals face minor additional benefits from transformation. Consequently, with government regulation, wholly foreign-owned hospitals choose to enter the China's healthcare sector, while public hospitals retain their existing operational model.

Scenario 3: when *R*_1_+*S*_1_<*C*_1_−*C*_5_, *C*_2_−*R*_2__<*S*_2_+*C*3_, and *C*_4_+*R*_3_<*S*_3_ hold, i.e., the overall benefits of wholly foreign-owned hospitals entering the China's healthcare sector are less than their costs, the costs of public hospital transformation are lower than the losses from not transform, and the social loss caused by government not regulation exceeds the sum of regulation administrative costs and financial subsidies to public hospitals. The corresponding stable equilibrium point is *E*_7_(0, 1, 1), reflecting the evolutionarily stable strategy combination as (not enter, not transform, and regulation). In this scenario, since the social loss from non-regulation outweighs the total costs of regulation (including financial subsidies), the government will adopt regulation. However, despite government regulation, wholly foreign-owned hospitals' entry costs exceed their overall benefits, while public hospitals face significant losses if they avoid transformation. Consequently, under governmental regulation, wholly foreign-owned hospitals opt against entering the China's healthcare sector, whereas public hospitals choose to transform their operational models.

Scenario 4: when *C*_1_<*R*_1_+*C*_5_+*S*_1_, *C*_2_−*R*_2_−*R*_3_<*C*_3_+*S*_2_, and *C*_4_+*R*_3_−*R*_4_<*S*_3_ hold, i.e., the overall benefits of wholly foreign-owned hospitals entering the China's healthcare sector exceed their costs, the costs of public hospital transform are lower than the losses from not transform, and the social loss caused by government not regulation surpasses the sum of regulatory administrative costs and financial subsidies to public hospitals. The corresponding stable equilibrium point is *E*_8_(1, 1, 1), representing the evolutionarily stable strategy combination (enter, transform, and regulation). In this scenario, the government adopts regulation because the social loss from not regulation outweighs the total costs of regulation (including financial subsidies). Meanwhile, wholly foreign-owned hospitals enter benefits under government regulation exceed their costs, and public hospitals face significant losses if they do not transform. Consequently, with regulation frameworks in place, wholly foreign-owned hospitals choose to enter the China's healthcare sector, while public hospitals opt to transform their operational models.

In summary, under government regulation and current conditions, the benefits of the game depend on the costs, losses, government financial subsidies, and strategies of stakeholders. The decision of wholly foreign-owned hospitals to enter the China's healthcare sector depends on their overall benefits, while their strategic choices are also influenced by government regulation. The decision of public hospitals to transform is significantly influenced by government financial support; due to their unique public welfare nature, they exhibit lower sensitivity to financial losses and gains. Government regulatory strategies require comprehensive considerations of administrative costs, fiscal resources, and other factors. However, promoting the entry of wholly foreign-owned hospitals and encouraging public hospital transformation are not merely economic issues but also societal challenges. To enhance patient healthcare experiences and maintain social stability, governments must implement regulatory policies that balance market openness with systemic sustainability.

## 5 Simulation analysis

### 5.1 Simulation analysis data

This study utilizes MATLAB R2024a to simulate and analyze the evolutionary game behavior of three participants, wholly foreign-owned hospitals, public hospitals, and government in the process of opening up China's healthcare sector. For the system to converge to the equilibrium state *S*_8_(1, 1, 1), the following stability conditions must be satisfied: *C*_1_−*R*_1_−*S*_1_−*C*_5_ < 0, *C*_2_−*C*_3_−*R*_2_−*S*_2_−*R*_3_ < 0, and *C*_4_+*R*_3_−*R*_4_−*S*_3_ < 0. To analyze the impact of individual parameters on equilibrium stabilization strategies, all non-target variables are held constant at values corresponding to the equilibrium state. This methodological ensures unambiguous attribution of behavioral changes in the tripartite evolutionary game to specific parameter variations. Concurrently, China's ongoing healthcare reform recognizes that openness is an important addition to the healthcare sector (10). Government agencies, increasingly cognizant of both the socioeconomic benefits (e.g., resource diversification, service innovation) and challenges (e.g., regulatory complexity, security risks) inherent in this transform, are adopting proactive governance frameworks to balance healthcare sector openness with systemic stability (51). Let *x* = 0.5, *y* = 0.5, and*z* = 0.6 (51), other parameters, such as the strength of incentives, subsidies, and penalties, are referenced in government documents and previous research results. The initial value of each parameter is set as follows: *C*_1_ = 11 (10), *C*_4_ = 5, *C*_2_ = 35 (50), *C*_3_ = 4 (10), *C*_5_ = 2 (57), *R*_1_ = 7, *R*_2_ = 30 (62), *R*_3_ = 3 (40), *S*_1_ = 3 (63), *S*_2_ = 3, *S*_2_ = 8 (23).

### 5.2 Simulation analysis of the initial values affecting evolution

**Figure 1:**
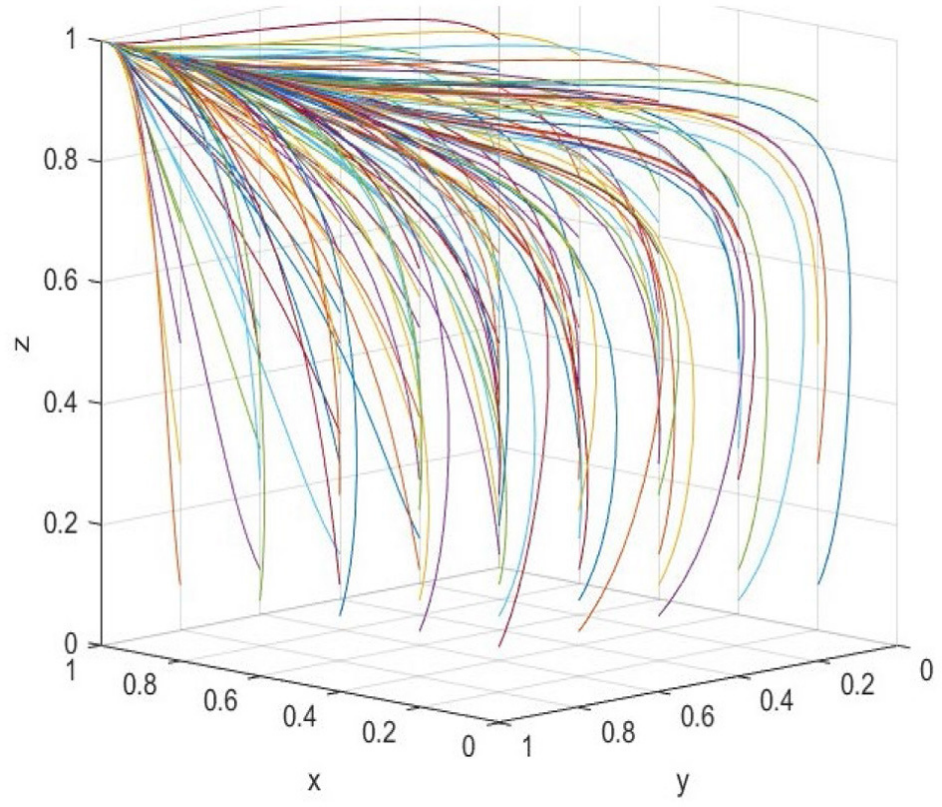
Random initial values influence.

To analyze the influence of different initial values of each of the three participants on the overall evolution path, the initial values of wholly foreign-owned hospitals, public hospitals, and government are selected cyclically in the interval [0,1] with a unit of 0.2. The resulting evolution is presented in Figure 1. It can be found that although different initial values lead to different evolution rates, the system will reach the *S*_8_(1, 1, 1). While most evolutionary trajectories exhibit smooth progression, the *z*-axis trajectory demonstrates a distinct inflection point during its evolution. Notably, the inflection diminishes as the initial values of *x* and *y* increase, ultimately converging toward the equilibrium state *S*_8_(1, 1, 1). This phenomenon underscores the interdependence of the tripartite participants' initial strategies, revealing that strategic alignment among wholly foreign-owned hospitals, public hospitals, and government critically modulates the system's path-dependent convergence behavior.

**Figure 2:**
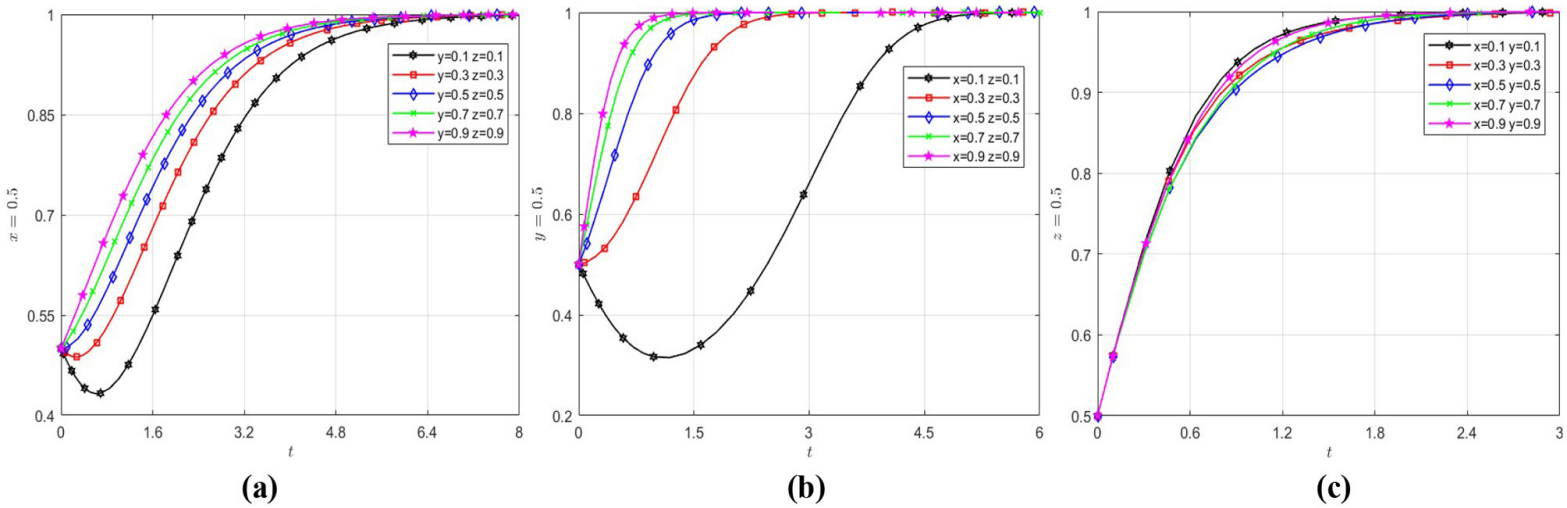
Effect of initial values on evolutionary paths. **(a)** The wholly foreign-owned hospitals evolution path diagram under different initial strategy choice. **(b)** The public hospitals evolution path diagram under different initial strategy choice. **(c)** The government evolution path diagram under different initial strategy choice.

The correlation of initial strategies among the three parties was tested by fixing one party's initial strategy selection probability at 0.5 and varying the others across five levels (0.1, 0.3, 0.5, 0.7, and 0.9), to analyse the relationship between the initial strategy choice probability of one party and the remaining two parties. The results were shown in Figure 2.

As shown in Figure 2a, wholly foreign-owned hospitals converge to the “enter China” strategy. Initially, due to low government regulation intensity, they trend toward not entry strategies early in the game. However, as China's healthcare sector opens further and regulatory policies reduce entry barriers, they ultimately adopt the entry strategy. As shown in Figure 2b, public hospitals ultimately adopt a “transformation” strategy. Initially, due to low entry rates of wholly foreign-owned hospitals and limited government financial subsidies, public hospitals resist transformation. However, as wholly foreign-owned hospitals gradually enter China's healthcare sector and government policies encourage high-quality healthcare sector, public hospitals shift decisively toward transformation. Figure 2c shows that the government evolves to regulation most rapidly when the probability of entry of wholly foreign-owned hospitals and transformation of public hospitals is low, reflecting the government's proactive attitude in guiding the healthcare sector toward quality and diversification.

### 5.3 Factors influencing the strategic choice of wholly foreign-owned hospitals

**Figure 3:**
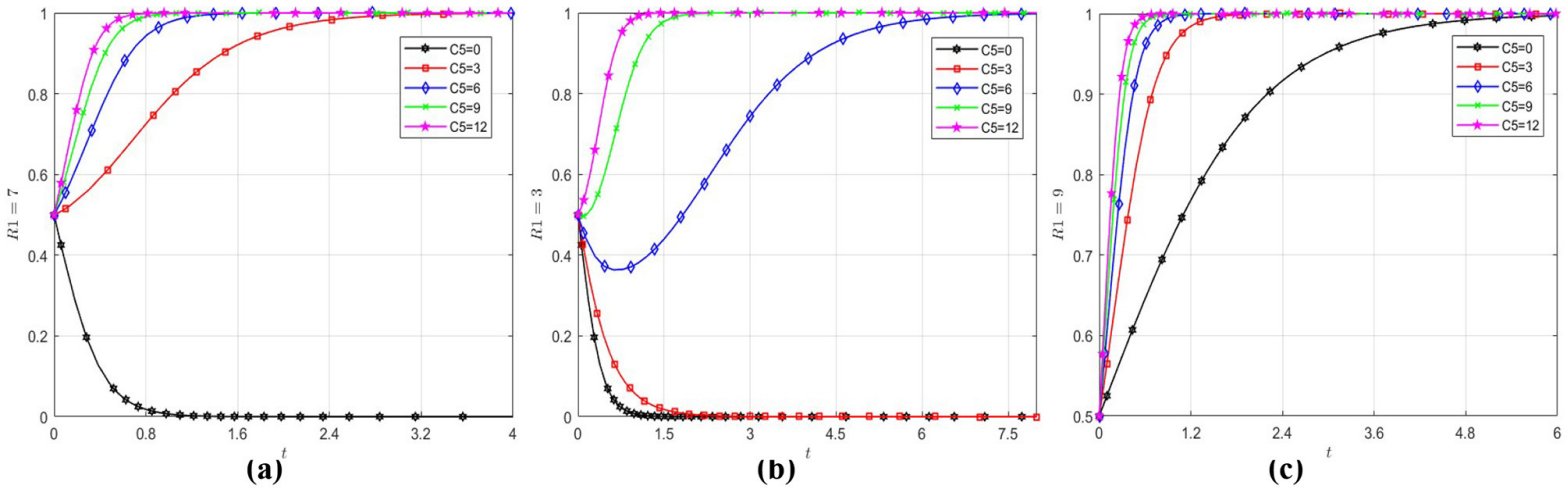
Impact of *R*_1_ and *C*_5_ on wholly foreign-owned hospitals strategic choice. **(a)** When R_1_ = 3, the wholly foreign-owned hospitals evolution path diagram under different C5. **(b)** When R_1_ = 7, the wholly foreign-owned hospitals evolution path diagram under different C5. **(c)** When R_1_ = 9, the wholly foreign-owned hospitals evolution path diagram under different C5.

Under the initial parameter settings, the variables *x, y* and *z* are initialized to (0.5, 0.5, 0.5) with *R*_1_and *C*_5_ varied to evaluate their impact on strategic choices for wholly foreign-owned hospitals. Figure 3 summarizes the findings. As evidenced in Figures 3a–c, when the intensity of government policy support remains constant, enhanced economic benefits significantly incentivize wholly foreign-owned hospitals to enter China's healthcare sector. Notably, when economic benefits reach a sufficiently high level, government policy support ceases to influence the strategic choices of wholly foreign-owned hospitals. Under stable economic conditions, government policy support accelerates the evolutionary rate of wholly foreign-owned hospitals toward convergence at 1. However, once policy support intensity surpasses a critical threshold, its promoting effect on the evolutionary trajectory of wholly foreign wholly-owned hospitals toward 1 gradually weakens.

An analysis of practical demands within China's healthcare sector indicates that the sector is undergoing accelerated development. Given sustained economic growth and rising household income levels, public demand for medical and wellness services has demonstrated persistent intensification. Wholly foreign-owned hospitals, capable of delivering more personalized and premium-tier medical services, are positioned to fulfill the demand for high-end healthcare among specific high-income demographic segments, consequently sustaining revenue streams at robust levels. Consequently, wholly foreign-owned hospitals demonstrate strong incentives to enter China's healthcare sector (64). Appropriate policy support from the government could accelerate the evolution of wholly foreign-owned hospitals toward entry into the China's healthcare sector, though such policy support is subject to diminishing marginal effects.

### 5.4 Factors influencing the strategic choice of public hospitals

**Figure 4:**
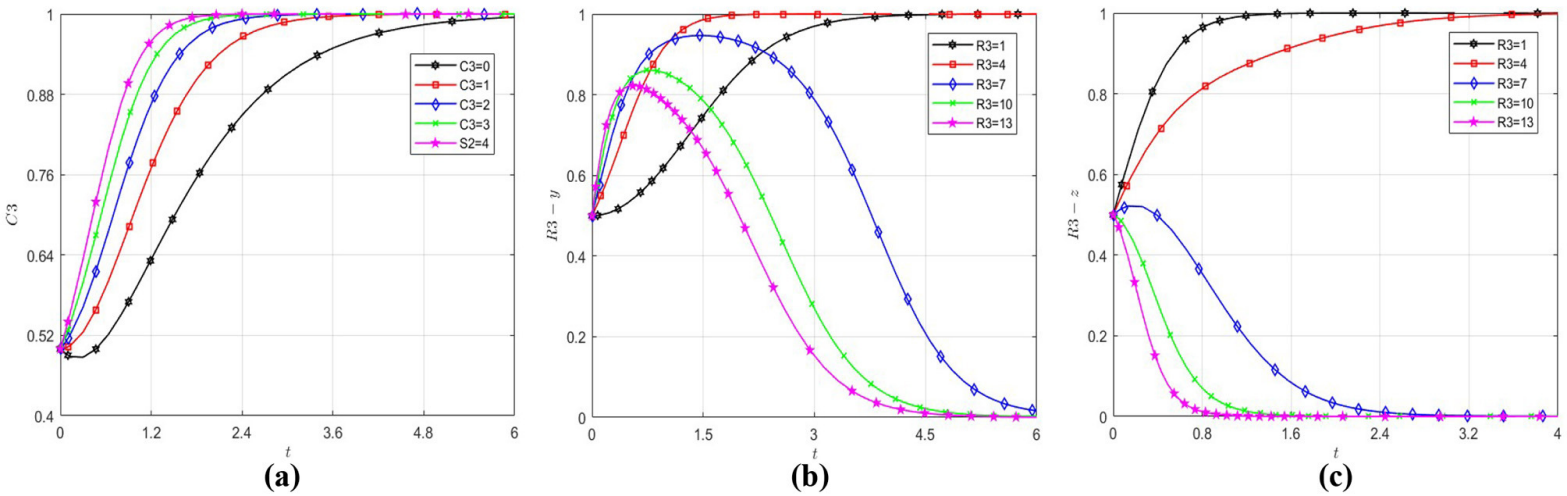
Influence of different factors on public hospitals strategic choice. **(a)** The public hospitals evolution path diagram under different C3. **(b)** The public hospitals evolution path diagram under different R3. **(c)** The government evolution path diagram under different R3.

Similarly, by setting the initial values of *x, y* and *z* to (0.5, 0.5, 0.5) and selecting varying values of *C*_3_ and *R*_3_, the analysis explores the influence of losses and financial subsidies on public hospitals' strategic choice, with the outcomes depicted in Figure 4. From Figure 4a, it can be observed that although the convergence rate of public hospitals toward to 1 increase with the magnitude of losses incurred by the entry of wholly foreign-owned hospitals into the China's healthcare sector, such losses are not the determining factor in their strategic choice to transform. Even when the loss parameter is set to zero (*C*_3_ = 0), public hospitals initially exhibit reluctance to adopt transformation strategies but ultimately converge toward transformation over time. Figure 4b demonstrates that there is a significant inverted U-shaped effect of financial subsidies on the choice of strategies by public hospitals. Prior to reaching a critical threshold of financial subsidies, the convergence rate of public hospitals toward to 1 increase with the escalation of financial subsidy levels. However, when subsidies exceed this critical threshold, with the increase of financial subsidy, although strategic choices initially evolve rapidly toward to 1, but ultimately stabilizes at 0. The reason is demonstrated in Figure 4c, the government's propensity to implement regulatory measures exhibits a diminishing response to escalating financial subsidies. Beyond a critical threshold, increased financial subsidy allocations trigger rapid convergence toward 0, coinciding with public hospitals' accelerated convergence to 0. This convergence mechanism underscores a robust causal linkage between the government's financial subsidies and the transformation of public hospitals.

The simulation results show that the strategic choice of public hospitals is strongly influenced by the strategic choice of the government. This reason originates from public hospitals' inherent public welfare-oriented institutional mandate. As non-profit entities with strictly regulated revenue mechanisms under state control, their transformational pathways become operationally contingent upon financial subsidies allocations from government financial subsidies (63). While heightened financial subsidies may accelerate the transformation propensity of public hospitals, such elevated subsidy levels prove unsustainable for governments in the long term (23). When governments lean toward non-regulatory approaches, public hospitals tend to regress toward non-transformation due to diminished financial subsidies, reflecting a codependent dynamic between government interventions and public hospitals reform trajectories (10).

### 5.5 Factors influencing the strategic choice of government

**Figure 5:**
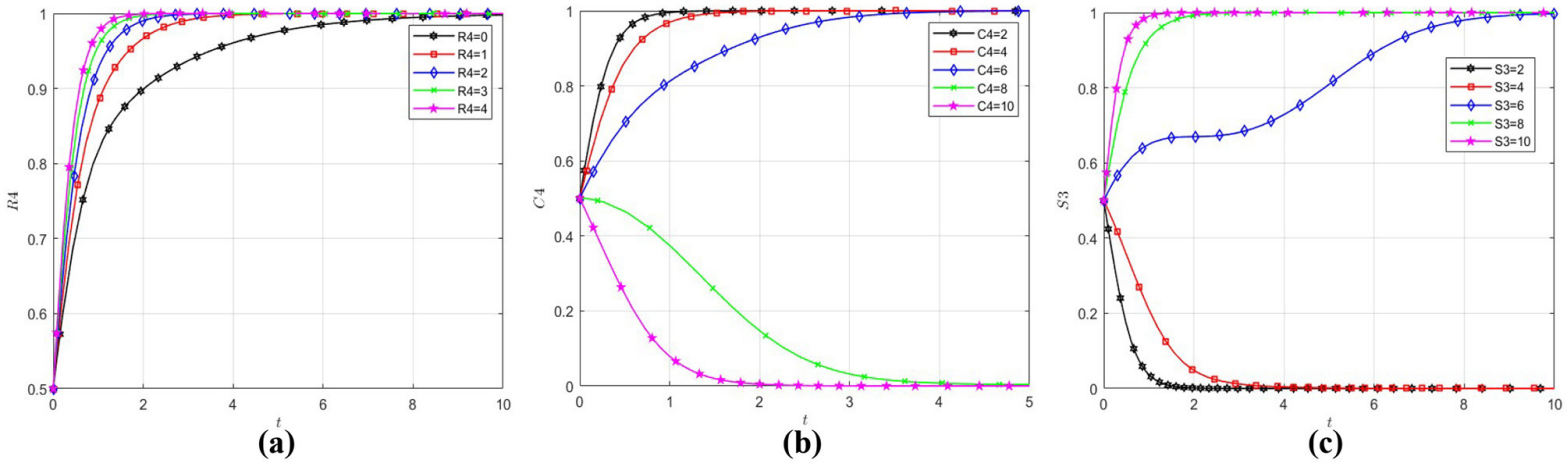
Influence of different factors on government strategic choice. **(a)** The government evolution path diagram under different R4. **(b)** The government evolution path diagram under different C4. **(c)** The government evolution path diagram under different S3.

Following the same methodology, with initial values of *x, y* and *z* set to (0.5, 0.5, 0.5) and by varying the parameters *R*_4_, *C*_4_, and *S*_3_, the analysis investigates determinants of government's strategic choice. The resultant behavioral patterns are quantified in Figure 5. As demonstrated in Figure 5a, regardless of the magnitude of parameter *R*_4_, the government's strategic choice orientation consistently converges toward regulation. This validates that the government's strategic choice is not effected by social benefits. From Figure 5b, it can be observed that the critical value of the government's cost of regulation is between 6 and 8, and when the government's cost of regulation exceeds this critical value, it will evolve to 0. The analysis of Figure 5c identifies a critical threshold range (4–6) for social loss. Beyond this interval, the government exhibits accelerated convergence toward regulation, demonstrating that social pressures dominate the government's strategic choice when social loss exceeds tolerance thresholds.

Research shows social losses and cost of regulation mainly effect government strategic choice, but social benefits have little effect. This cost-benefit asymmetry underscores the predominance of loss aversion over gain maximization in Chinese healthcare sector governance frameworks. This demonstrates that the government strategic choice inherently embodies a strong public welfare orientation. The formulation of public policies is fundamentally driven by the imperative to maintain the stability of the economy and society (65), guided by the developmental needs of society. It necessitates that efficiency improvements in public services achieve equilibrium between policy provisions and citizen demands (66).

## 6 Discussion and suggestions

### 6.1 Conclusions

This study systematically explores the interactions among wholly foreign-owned hospitals, public hospitals, and the government by constructing an evolutionary game model and conducting system dynamics simulations to analyze the behavioral characteristics of these stakeholders. It provides decision-making insights for optimizing their development under China's ongoing expansion of healthcare sector openness. Key findings from the simulations include: Benefits are the critical determinant of strategic choices for foreign-funded hospitals. Government policy support acts as a catalyst, though its effect diminishes as benefits increase. The entry of wholly foreign-owned hospitals significantly accelerates the transformation efficiency of public hospitals, but is not the decisive factor. Government fiscal subsidies emerge as the determining factor for public hospital reform. For the government, costs and losses dominate strategic choices, while benefits play a promoting role.

Therefore, government regulation can encourage the entry of wholly foreign-owned hospitals into the China's healthcare sector by lowering barriers such as market entry thresholds, while requiring public hospitals to transform through financial subsidies. However, it is critical to ensure that regulatory intensity remains within a reasonable range. This can avoid diminishing marginal effects in supporting wholly foreign-owned hospitals and prevent financial subsidies to public hospitals from becoming a long-term financial burden. Additionally, governments prioritize their reputational capital; the reputational damage caused by not regulation would further incentivize policymakers to adopt proactive regulation policies. By balancing these considerations, regulation can effectively foster healthcare sector openness and institutional reform while safeguarding public interests and financial sustainability.

### 6.2 Discussion

Many scholars agree that the demand of the public and patients for a high-quality healthcare sector and diversified medical services is the fundamental driving force that prompts the government to continuously open up the healthcare sector and strongly support the transformation of public hospitals (67). However, previous studies have mainly focused on exploring the advantages of wholly foreign-owned hospitals entering the China's healthcare sector, or the necessity and key aspects of the transformation of public hospitals. Moreover, previous studies have mostly regarded government support as a key factor influencing the entry of wholly foreign-owned hospitals into the China's healthcare sector. However, this study further discovers that there is a law of diminishing marginal returns in government support. It shows that in the past decade (2015–2023), the continuous growth of China's healthcare sector (China's per capita healthcare expenditure increased from about 1,000–2,460 yuan) is the decisive factor driving the entry of foreign-funded hospitals (62), and the government only needs to provide an open market environment and appropriate policy support.

Most scholars believe that the presence of wholly foreign-owned hospitals can further promote the transformation of public hospitals through competitive pressure (10). However, this study finds that public hospitals are not sensitive to the impact of losses. The reason is that due to the inherent public welfare and non-profit nature of public hospitals and the existence of a fixed consumer group, in practice, they lack a strong internal motivation for transformation (9). The government must provide targeted financial subsidies to promote their reform, and the scale of financial support is directly proportional to the degree of transformation of public hospitals.

From the government's perspective, for wholly foreign-owned hospitals, the government only needs to maintain an open policy framework and reasonable supervision. For public hospitals, financial subsidies are still crucial, but the intensity must be strategically adjusted to ensure the sustainability of long-term financial subsidies.

### 6.3 Suggestions

Based on the above conclusions and the reality of development practice, the following countermeasures are proposed:

(1) Continuously strengthen market openness and expand foreign hospital access. To enhance healthcare quality, foster market competition, and improve patient experiences, governments should establish clearer and more comprehensive guidelines for wholly foreign-owned hospital entry. Developing transparent operational and regulatory standards and streamlining approval processes to reduce entry barriers and operational costs. Defining specific scopes of medical service access and permitting drugs aligned with international standards but distinct from China's standards. Allowing wholly foreign-owned hospitals to adopt diverse insurance models and fee structures while ensuring equal treatment in hiring foreign medical professionals or overseas specialists.
(2) Reform financial subsidy mechanisms to drive high-quality public hospital transformation. Given financial subsidy limitations, governments should open healthcare investment to social capital, channeling private funds into public hospitals to alleviate financial pressure and enable service quality upgrades. Governments must strengthen their leadership in upholding the non-profit nature and public welfare mandate of public hospitals. This includes promoting the establishment of integrated urban healthcare alliances anchored by high-performing public hospitals, ensuring their pivotal role in guaranteeing equitable access to basic medical and health services.
(3) The government should transition from extensive management to refined management. Governments should leverage digitalization and AI to reduce regulatory costs and enhance oversight precision. Expanding access to high-quality medical resources and promoting regional equity in healthcare distribution. Utilizing big data analytics to monitor hospital input-output efficiency, incentivizing cost reduction, and operational optimization to lower patient burdens and improve satisfaction. Establishing independent third-party evaluation bodies to ensure market vitality, foster innovation, and meet diverse patient needs. This framework balances market openness with institutional accountability, ensuring sustainable healthcare modernization aligned with public welfare goals.

In conclusion, achieving high-quality development in China's healthcare sector necessitates collaborative efforts among wholly foreign-owned hospitals, public hospitals, and the government. Through policy support, fiscal measures, and optimization of the business environment, China can comprehensively introduce high-caliber international medical resources, drive the high-quality transformation of public hospitals, and ultimately meet the multi-tiered and diverse healthcare demands of its population.

However, in real-world scenarios, the strategic interactions among these three stakeholders transcend bounded rationality, operating within a dynamic landscape shaped by multidimensional uncertainties. Cognitive biases, external disruptions, and evolving environmental pressures collectively exert non-linear impacts on decision-making trajectories. Future research should prioritize enhancing game-theoretic models by integrating adaptive frameworks that simulate behavioral heterogeneity, stochastic disturbances, and time-dependent feedback loops. This advancement would enable a granular exploration of how emergent complexities, such as policy volatility, resource constraints, and shifting stakeholder priorities, reshape equilibrium outcomes and long-term system resilience.

## Data Availability

The original contributions presented in the study are included in the article/supplementary material, further inquiries can be directed to the corresponding authors.

## References

[1] Chen J Lin Z Li L Li J Wang Y Pan Y. Ten years of China's new healthcare reform: a longitudinal study on changes in health resources. BMC Public Health. (2021) 21:2272. 10.1186/s12889-021-12248-9 34903184 PMC8670033

[2] Yip W Fu H Chen A Zhai T Jian W Xu D. 10 years of health-care reform in China: progress and gaps in universal health coverage. Lancet. (2019) 394:1192–204. 10.1016/S0140-6736(19)32136-1 31571602

[3] Jakovljevic M Lamnisos D Westerman R Chattu VK Cerda A. Correction to: future health spending forecast in leading emerging BRICS markets in 2030: health policy implications. Health Res Policy Syst. (2022) 20:30. 10.1186/s12961-022-00836-z 35300693 PMC8932142

[4] Xinhua News Agency. Decision of the Central Committee of the Communist Party of China on Further Deepening Reform and Promoting Chinese path to modernization (2024). Available online at: https://www.gov.cn/zhengce/202407/content_6963770.htm (accessed December 6, 2024).

[5] National Health Commission of the People's Republic of China. Working Plan for Expanding the Pilot Project in the Domain of wholly Foreign-Owned Hospitals (2024). Available online at: https://www.gov.cn/zhengce/zhengceku/202411/content_6990279.htm (accessed December 14, 2024).

[6] Shao Q Wang L Zhang W Zou M Ma Z Wang F. Research on the application of diversified medical service system to support the high-quality development of public hospitals. Chin Hosp. (2025) 29:25–8. 10.19660/j.issn.1671-0592.2025.2.06

[7] Sahoo PM Rout HS Jakovljevic M. Future health expenditure in the BRICS countries: a forecasting analysis for 2035. Global Health. (2023) 19:49. 10.1186/s12992-023-00947-4 37434257 PMC10334532

[8] Jakovljevic M. Comparison of historical medical spending patterns among the BRICS and G7. J Med Econ. (2015) 19:1–10. 10.3111/13696998.2015.1093493 26366470

[9] Zhou M. Deepening the reform of public hospitals guided by public welfare. Guangming Daily (2024). Available online at: http://www.qstheory.cn/qshyjx/2024-06/03/c_1130155936.htm (accessed December 27, 2024).

[10] Wu J Zhao W Bie R Miao D. Study on the explanatory framework of public hospital scale growth in China from the perspective of new institutional economics. Chin Hosp Manage. (2024) 44:13–6.

[11] Zhu N. Deepening the reform of public hospitals guided by public welfare. Legal Daily (2024).

[12] Pan J Qin X Hsieh C. Is the pro-competition policy an effective solution for China's public hospital reform? Health Econ Policy Law. (2016) 11:337–57. 10.1017/S1744133116000220 27346712

[13] Wang Z Zhu F. Research report on China's ten-year healthcare reform (2009-2019). Institute of Economics Chinese Academy of Social Sciences (2019). Available online at: http://ie.cass.cn/kygz/zzxz/202409/W020240925398274508971.pdf (accessed January 13, 2025).

[14] Kabir KMA Tanimoto J. Modelling and analyzing the coexistence of dual dilemmas in the proactive vaccination game and retroactive treatment game in epidemic viral dynamics. Proc Royal Soc A Math Phys Eng Sci. (2019) 475:20190484. 10.1098/rspa.2019.0484 31892836 PMC6936617

[15] Zhao Y. Wholly foreign-owned hospitals seize the Chinese market. China Today (2025). Available online at: http://www.chinatoday.com.cn/zw2018/bktg/202503/t20250311_800395056.html

[16] Sun H. The first wholly foreign-owned tertiary hospital has arrived. News China (2025). Available online at: https://news.inewsweek.cn/society/2025-02-25/24543.shtml

[17] Lu L Pan J. Does hospital competition lead to medical equipment expansion? Evidence on the medical arms race. Health Care Manage Sci. (2021). 24:582–96. 10.1007/s10729-020-09529-x 33411086

[18] Luo L Wang Y. Insights from the ‘Changgeng model' and the ‘Mayo model' on performance management reform in public hospitals. Legal Syst Soc. (2018) 176–7. 10.19387/j.cnki.1009-0592.2018.08.322

[19] Fu H. Analysis and optimization of external regulatory policies for large public hospitals. Chin Hosp. (2023) 27:40–2. 10.19660/j.issn.1671-0592.2023.04.11

[20] Giammanco MD Gitto L. Health expenditure and FDI in Europe. Econ Anal Policy. (2019) 62:255–67. 10.1016/j.eap.2019.04.001

[21] de Vries N Boone A Godderis L Bouman J Szemik S. The race to retain healthcare workers: a systematic review on factors that impact retention of nurses and physicians in hospitals. Inquiry. (2023) 60:469580231159318. 10.1177/00469580231159318 36912131 PMC10014988

[22] Yuan L Cao J Wang D Yu D Liu G Qian Z. Regional disparities and influencing factors of high-quality medical resources distribution in China. Int J Equity Health. (2023) 22:8. 10.1186/s12939-023-01825-6 36627636 PMC9832614

[23] Wu Q Wang T. An evolutionary game analysis on the management of medical quality homogeneity in tight county medical community. Chin Health Standard Manage. (2024) 15:87–92. 10.3969/j.issn.1674-9316.2024.09.021

[24] Behr CL Hull P Hsu J Newhouse JP Fung V. Geographic access to federally qualified health centers before and after the affordable care act. BMC Health Serv Res. (2022) 22:385. 10.1186/s12913-022-07685-0 35321700 PMC8942056

[25] Deng R Su Y. An overview of the development and research on the financial and accounting supervision of public hospitals in China. Chin Health Econ. (2024) 43:81–6.

[26] Saghafian S. Drivers, adaptations, and public impacts of hospital closures: implications for policy. Front Public Health. (2024) 12:1415033. 10.3389/fpubh.2024.1415033 39193198 PMC11347414

[27] DJ BY ZL SX. Construction and practical exploration of operation and management mode in a large comprehensive public hospital. Chin Hosp. (2025) 29:77–80. 10.19660/j.issn.1671-0592.2025.1.17

[28] Wang S Guo Z Zhao Y Zheng L Hu J Jiang S. Research on the evolution and development trend of operation management of public hospitals in China. Chin Hosp Manage. (2025) 45:15–7.

[29] Jakovljevic M Chang H Pan J Guo C Hui J Hu H. Successes and challenges of China's health care reform: a four-decade perspective spanning 1985-2023. Cost Eff Resour Alloc. (2023) 21:59. 10.1186/s12962-023-00461-9 37649062 PMC10469830

[30] Han G Zheng X Zeng M Wang C Wang F. The coping strategies of social medical institutions under the background of promoting Sanming healthcare reform nationwide. Chin Health Econ. (2025) 44:15–7.

[31] He Y Dou G Huang Q Zhang X Ye Y Qian M. Does the leading pharmaceutical reform in China really solve the issue of overly expensive healthcare services? Evidence from an empirical study. PLoS ONE. (2018) 13:e0190320. 10.1371/journal.pone.0190320 29338038 PMC5770029

[32] Hu L Fu M Wushouer H Ni B Li H Guan X. The impact of Sanming healthcare reform on antibiotic appropriate use in county hospitals in China. Front Public Health. (2022) 10:936719. 10.3389/fpubh.2022.936719 35832279 PMC9271699

[33] Chen H Ding X Jiang M. Research on internal and external linkage mechanism to promote the high- quality development of clinical specialties in public hospitals. Chin Hosp Manage. (2025) 45:20–4.

[34] Wang Z. An analysis of high-quality development strategies for clinical disciplines in public hospitals. Mod Hosp Manage. (2024) 22:5–8. 10.3969/j.issn.1672-4232.2024.05.002

[35] Feng L Pan L Fan X Wang Z Zhong L. Research on the integrated management model of multiple hospital areas in public hospitals from the perspective of 7s theory. Chin Hosp. (2025) 29:40–4. 10.19660/j.issn.1671-0592.2025.2.09

[36] Meskó B Topol EJ. The imperative for regulatory oversight of large language models (or generative AI) in healthcare. NPJ Digit Med. (2023) 6:120–6. 10.1038/s41746-023-00873-0 37414860 PMC10326069

[37] Pan L Xiao K Zhu H Luo L. The impacts of public hospital comprehensive reform policies on hospital medicine cost, revenues and healthcare expenditures 2014-2019: an analysis of 103 tertiary public hospitals in China. Front Health Serv. (2023) 3:1079370. 10.3389/frhs.2023.1079370 36926494 PMC10012765

[38] Guo B Feng W Cai H Lin J. Influence of public hospital reform on public health: evidence from a quasi-natural experiment in China. Front Public Health. (2023) 11:1104328. 10.3389/fpubh.2023.1104328 37033016 PMC10079936

[39] Nundoochan A. Improving public hospital efficiency and fiscal space implications: the case of Mauritius. Int J Equity Health. (2020) 19:152. 10.1186/s12939-020-01262-9 32887629 PMC7473700

[40] Ye S. Improve the government compensation mechanism for public hospitals. Macroecon Manage. (2021) 4:48–54. 10.19709/j.cnki.11-3199/f.2021.04.010

[41] Zhang M Hu Y Dai D Zhang Y Xu D. Research on policy characteristics and advancement strategies for improving patient healthcare experiences from the perspective of campaign-style governance. Chin Hosp. (2025) 29:32–6. 10.19660/j.issn.1671-0592.2025.3.07

[42] Wang R He D Fu Y Luo Y Xing Q Wang H. Discussion on building a medical artificial intelligence technology assessment system suitable for Chinese National Condition. Chin Health Econ. (2024) 43:38–43.

[43] Li M Liu M Qin H Zhu Y Liu R. Research on the innovation mechanism of hospital-enterprise scientific research cooperation based on evolutionary game. Chin Hosp Manage. (2023) 43:60–3.

[44] Tian S Tong M Li X. Fiscal expenditure, government competition, and healthcare service level: an empirical analysis based on the provincial panel entropy Tobit model. J Yun Univ Fin Econ. (2022) 38:19–36. 10.16537/j.cnki.jynufe.000807

[45] Habib MA Kabir KMA Tanimoto J. Evolutionary game analysis for sustainable environment under two power generation systems. Evergreen. (2022) 9:326–44. 10.5109/4793672

[46] Yin L Li S Gao F. Equilibrium stability of asymmetric evolutionary games of multi-agent systems with multiple groups in open electricity market. IEEE Access. (2020) 8:28970–8. 10.1109/ACCESS.2020.2972387

[47] Tanimoto J. Evolutionary game theory. In:Tanimoto J, editor. Evolutionary Games with Sociophysics: Analysis of Traffic Flow and Epidemics. Singapore: Springer Singapore (2018). p. 11–103. 10.1007/978-981-13-2769-8_2

[48] Alam M Kuga K Tanimoto J. Three-strategy and four-strategy model of vaccination game introducing an intermediate protecting measure. App Math Comput. (2019) 346:408–22. 10.1016/j.amc.2018.10.015

[49] Kabir KMA Tanimoto J. Dynamical behaviors for vaccination can suppress infectious disease – A game theoretical approach. Chaos Solitons Fract. (2019) 123:229–39. 10.1016/j.chaos.2019.04.010

[50] Chen S Ding Y. Study on the tripartite evolutionary game among hospitals, service providers and suppliers under SPD supply Chain management mode. Chin Health Econ. (2024) 43:75–80.

[51] Liang F Liu L Li H. Evolutionary game and simulation analysis of pharmaceutical enterprises' competitive behavior under the background of centralized bulk-buying. Econ Manage. (2023) 37:83–92. 10.3969/j.issn.1003-3890.2023.04.009

[52] Zhang Q Wang L Geng N Jiang Z. Evolutionary game analysis of medical information sharing based on the government regulation. Operat Res Manage Sci. (2020) 29:23–31. 10.12005/orms.2020.0004

[53] Guan X Zhang Z Chen Z Gao Y. The evolutionary game model of medical dispute under the regulation of the government. Syst Eng Theory Pract. (2019) 39:3151–62. 10.12011/1000-6788-2019-0168-12

[54] Zhu L Rong J. Three-party evolutionary game and simulation analysis of drug quality supervision under the government reward and punishment mechanism. Chin J Manage Sci. (2021) 29:55–67. 10.16381/j.cnki.issn1003-207x.2019.0481

[55] Ji G Wang Q Chang Q Fang Y Bi J Chen M. Evolutionary game analysis of government regulation on green innovation behavior decision-making of energy enterprises. Sustainability. (2024) 16:7542. 10.3390/su16177542

[56] Lu Y Wang J. The practice of foreign -domestic joint venture hospitals under the new medical policy. Modern Hospital. (2015) 15:1–3. 10.3969/j.issn.1671-332X.2015.08.001

[57] Yu L Liu H. Analysis of patient welfare effects of hospital competition: evidence from a quasi-natural experiment on public hospital reform. Res Financ Econ Issues. (2023) 4:115–29. 10.19654/j.cnki.cjwtyj.2023.04.009

[58] Onofrei M Vatamanu A Vintilă G Cigu E. Government health expenditure and public health outcomes: a comparative study among EU developing countries. Int J Environ Res Public Health. (2021) 18:10725. 10.3390/ijerph182010725 34682472 PMC8535729

[59] Xu J Li F Zhu B Qin Q Jin C. Research on the compensation mechanism reform in Shanghai public hospitals under the background of medical-pharmaceutical separation. Chin Health Econ. (2024) 43:35–9.

[60] Friedman D. Evolutionary economics goes mainstream: a review of the theory of learning in games. J Evolution Econ. (1998) 8:423–32. 10.1007/s001910050071

[61] Ritzberger K Weibull JW. Evolutionary selection in normal-form games. Econometrica. (1995) 63:1371–99. 10.2307/2171774

[62] National Health Commission of the People's Republic of China. 2023 China Health Statistics Yearbook. Beijing: China Union Medical College Press (2024).

[63] Zheng S. The research of competitive strategy of H Hospital. South China University of Technology (2023).

[64] Su X. Research on the improvement of diagnosis and treatment process in P foreign -funded Hospital. University of Electronic Science and Technology of China (2022).

[65] Lin Y. Serving the people: a study on the decision motivation of the Chinese government. Chinese Social Sciences Today (2021). Available online at: https://www.cssn.cn/skgz/bwyc/202208/t20220803_5464791.html (accessed December 11, 2024).

[66] Yan J Zhu C. The adjustment of Chinese public policies: a discussion on the value orientation and practice of ‘Putting the People at the Center'. Governance Studies. (2021) 37:29–40. 10.3969/j.issn.1007-9092.2021.05.004

[67] NHSA. Statistical Bulletin of National Medical Security Development in 2023 (2024). Available online at: https://www.nhsa.gov.cn/art/2024/7/25/art_7_13340.html (accessed January 25, 2025).

